# Injury of an aberrant internal carotid artery after myringotomy

**DOI:** 10.11604/pamj.2017.27.237.9092

**Published:** 2017-08-02

**Authors:** Aryé Weinberg, Andreas Eberhard Albers

**Affiliations:** 1 Prosper-Hospital, Department of Otorhinolaryngology, Head and Neck Surgery, Recklinghausen, Germany; 2 Charité Universitätsmedizin, Campus Benjamin Franklin, Department of Otorhinolaryngology, Head and Neck Surgery, Berlin, Germany

**Keywords:** Aberrant internal carotid, myringotomy, conductive hearing loss

## Image in medicine

A massive bleeding from the right ear, the nose and from the mouth occurred in a 46-year-old female after ambulatory myringotomy was performed in order to treat symptoms mimicking a persisting middle ear effusion with conductive hearing loss. The patient was directly admitted to our intensive care unit. The bleeding continued until the blood pressure was lowered to normal and the patient was positioned in an upright position. Otoscopy showed a pulsatile bleeding through a perforation of the tympanic membrane. Endoscopy of the epipharynx showed fresh blood coming from the eustachian tube. MRI (Panel A) and angiography (Panel B) showed an aberrant internal carotid artery on the right side in the petrousal part with a thorn-like protrusion at the side of injury followed by a decreased vessel-diameter directly below the injury (Panel A, B). No coincidental aneurysm or vascularised tumor were found. An aberrant internal carotid artery in the middle ear is rare. Without prior diagnosis routine myringotomy can cause life-threatening situations. If a bluish-red formation behind the tympanic membrane is seen combined with the symptoms of pulsatile tinnitus and conductive hearing loss, a vascular malformation in the middle ear should be suspected and imaging of the temporal bone should precede any intervention. To avoid puncture of aberrant vessels in the middle ear, a paracentesis should in suspicious cases rather be performed with a sickle knife especially and not with a lancet.

**Figure 1:**
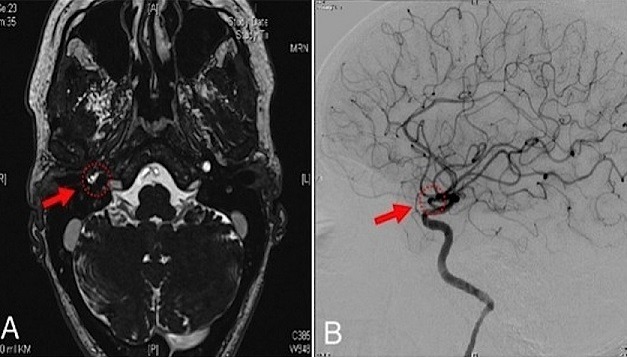
MRI (A) and angiography; (B) showing an aberrant internal carotid artery

